# Using the ACR CT accreditation phantom for routine image quality assurance on both CT and CBCT imaging systems in a radiotherapy environment

**DOI:** 10.1120/jacmp.v15i4.4835

**Published:** 2014-07-08

**Authors:** Maritza A. Hobson, Emilie T. Soisson, Stephen D. Davis, William Parker

**Affiliations:** ^1^ Department of Medical Physics McGill University Health Centre, Montréal, and Medical Physics Unit, McGill University Montréal QC Canada

**Keywords:** CBCT system, ACR, image quality, quality assurance

## Abstract

Image‐guided radiation therapy using cone‐beam computed tomography (CBCT) is becoming routine practice in modern radiation therapy. The purpose of this work was to develop an imaging QA program for CT and CBCT units in our department, based on the American College of Radiology (ACR) CT accreditation phantom. The phantom has four testing modules, permitting one to test CT number accuracy, slice width, low contrast resolution, image uniformity, in‐plane distance accuracy, and high‐contrast resolution reproducibly with suggested window/levels for image analysis. Additional tests for contrast‐to‐noise ratio (CNR) and noise were added using the polyethylene and acrylic plugs. Baseline values were obtained from CT simulator images acquired on a Phillips Brilliance Big Bore CT simulator and CBCT images acquired on three Varian CBCTs for the imaging protocols most used clinically. Images were then acquired quarterly over a period of two years. Images were exported via DICOM and analyzed manually using OsiriX. Baseline values were used to ensure that image quality remained consistent quarterly, and baselines were reset at any major maintenance or recalibration. Analysis of CT simulator images showed that image quality was within ACR guidelines for all tested scanning protocols. All three CBCT systems were unable to distinguish the low‐contrast resolution plugs and had the same high‐contrast resolution over all imaging protocols. Analysis of CBCT results over time determined a range of values that could be used to establish quantitative tolerance levels for image quality deterioration. While appropriate for the helical CT, the ACR phantom and guidelines could be modified to be more useful in evaluating CBCT systems. In addition, the observed values for the CT simulator were well within ACR tolerances.

PACS numbers: 87.57.Q‐, 87.57.qp, 87.57.C‐

## I. INTRODUCTION

Image‐guided radiation therapy (IGRT) using in‐room (or on‐board) cone‐beam computed tomography (CBCT) is becoming routine practice in modern radiation therapy. Image quality is important, as images acquired after treatment setup are used to ensure accurate localization of the target or to ensure that the target is in the planned position relative to the treatment beam. Image contrast must be adequate to resolve target structures of both high‐ and low‐contrast objects. Hounsfield unit (HU) fidelity is also important if the images will be used for dose calculation.

Tolerance values for the above‐mentioned image quality parameters are well established for CT and CT simulation.^1^, ^2^ However, image quality tolerances for CT cannot be used for CBCT because of the inherent differences in image quality due to the scattered photon component in CBCT systems and differences in reconstruction techniques resulting in a noisier image.^3^, ^4^ For routine image quality assurance (QA) of CBCT systems used for image guidance, most of the recommendations from the literature compare uniformity and CT number constancy/accuracy QA results to “baseline” QA results without providing quantitative tolerance levels^5^, ^6^, ^7^, ^8^, ^9^ or provide relatively loose tolerance levels.^10^, ^11^, ^12^ Most of the QA results and programs reported in the literature have used the Catphan phantom^5^, ^9^, ^10^, ^11^, ^12^, ^13^ (The Phantom Laboratories, Salem, NY), which is used for both acceptance testing and commissioning of most linac CBCT systems.

Therefore, the purpose of this work was to develop an imaging QA program with tight quantitative tolerance levels for the image quality parameters on imaging units capable of acquiring CT images in a radiotherapy department (i.e., both conventional CT and CBCT systems). In order to ensure image quality consistency with baseline values established during commissioning and acceptance testing, a routine imaging QA program, using the American College of Radiology (ACR) CT accreditation phantom (model 464, Gammex‐RMI, Middleton, WI, USA), was established. The ACR phantom has four testing modules that allow one to test CT number accuracy, slice thickness, low‐contrast resolution, image uniformity, in‐plane distance accuracy, and high‐contrast resolution. The ACR CT phantom was picked because it has well‐established image quality tests and tolerances for conventional CT systems^1^, ^14^ and is quite easy to set up. These tests and tolerances provide a starting point for developing a routine QA program for CT and CBCT systems in a radiotherapy department based on one phantom.

## II. MATERIALS AND METHODS

**Figure 1:**
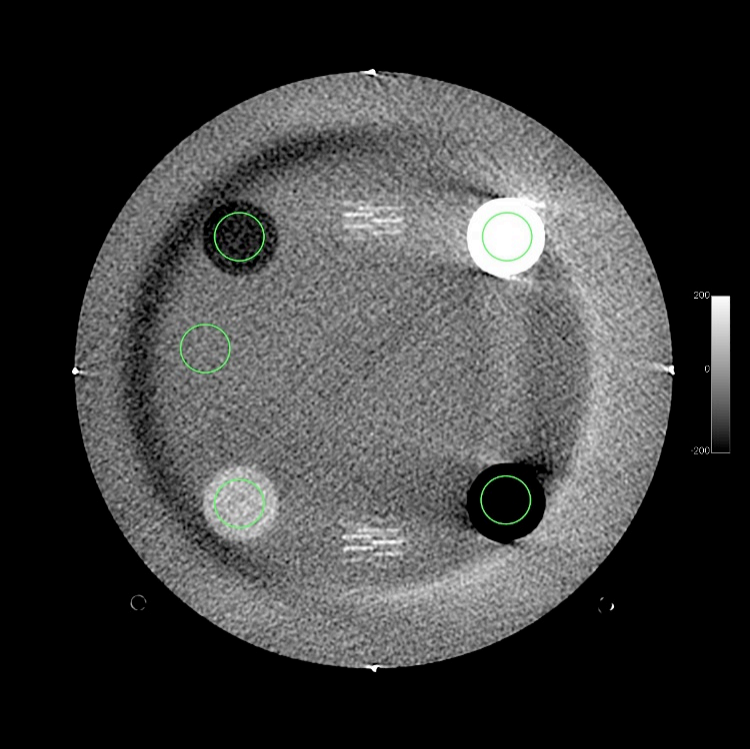
CBCT image of Module 1 of the ACR CT accreditation phantom, which is used for testing of HU fidelity and slice width. ROIs for HU determination with an area of approximately $200\;{\text{mm}}^{2}$ are overlaid over the different density plugs.

**Figure 2:**
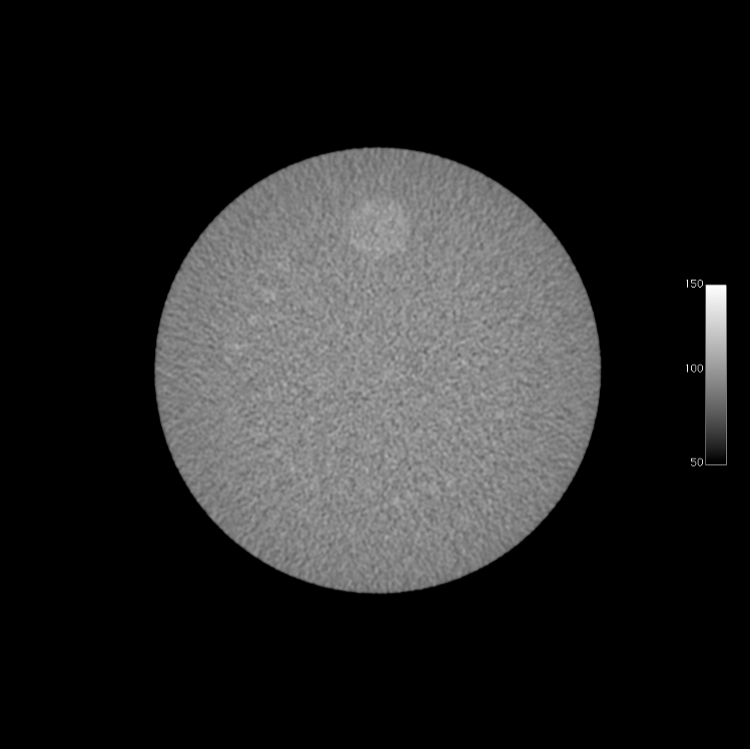
CT simulator image of Module 2 of the ACR CT accreditation phantom, which is used for determining low‐contrast resolution and CNR.

**Figure 3:**
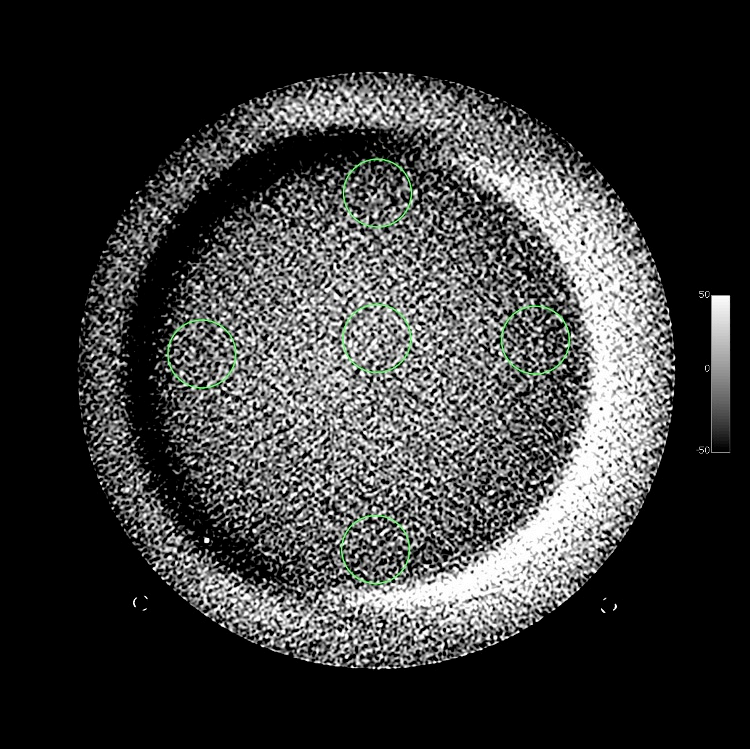
CBCT image of Module 3 of the ACR CT accreditation phantom, which is used for determining uniformity and in‐plane distance accuracy. ROIs for uniformity determination with an area of approximately $400\;{\text{mm}}^{2}$ are placed so that they are not within CBCT image artifacts. For in‐plane distance measurements, a different window and level may be needed to visualize the BBs.

**Figure 4:**
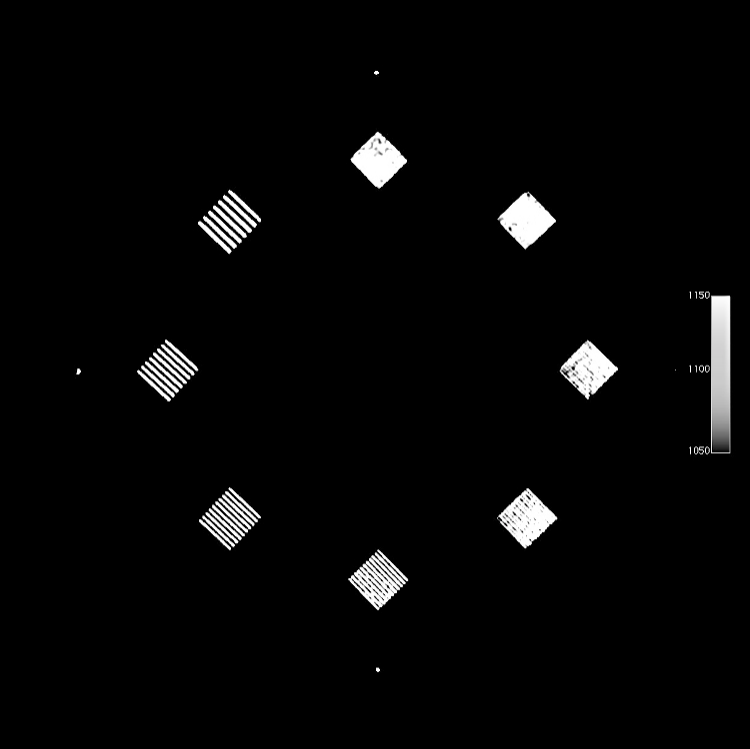
CBCT image of Module 4 of the ACR CT accreditation phantom, which is used for determining high‐contrast resolution.

The ACR phantom is 20 cm in diameter and has four modules with a length of 4 cm each for a total length of 16 cm.^(14)^ The first module allows for testing of HU fidelity and slice width, and a CBCT image of this module is shown in Fig. 1. The second module, Fig. 2, is used to determine low‐contrast resolution and CNR. The third module, Fig. 3, is used to test uniformity and in‐plane distance accuracy. The fourth module, Fig. 4, is used to determine high‐contrast resolution.

**Figure 5:**
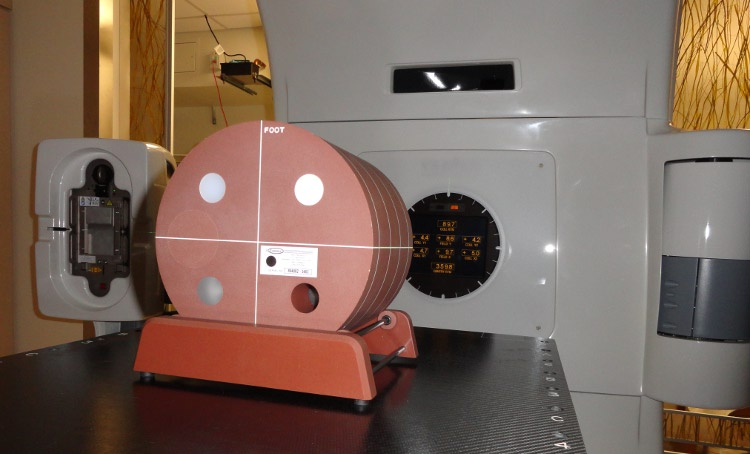
Picture of setup of ACR CT accreditation phantom on a linear accelerator.

Over a period of two years, CT and CBCT images of the ACR phantom were acquired quarterly, as well as after any major maintenance and repair. For both CBCT and conventional CT simulator images, the phantom was aligned with the scribed lines on the top and side of the phantom using the coronal and sagittal lasers. A photo of the setup on a linac is shown in Fig. 5. The phantom was adjusted longitudinally on the couch so that the axial laser was in the center of the phantom. This positioning of the phantom only requires one acquired CT or CBCT image volume per protocol used, making acquisition of images faster. If the phantom appeared to be misaligned on initial imaging, the phantom position was adjusted and images were reacquired.

CT simulator images of the ACR phantom were acquired on a Brilliance Big Bore CT scanner (Philips Medical Systems, Bothell, WA) with the scanning protocols most used clinically. The parameters for these protocols are given in Table 1. Note that these are not the parameters that are recommended for ACR accreditation scans.

CBCT images of the ACR phantom were acquired on three linear accelerators (two Varian Clinac 21EXs and one Varian Novalis Tx) (Varian Medical Systems, Palo Alto, CA) using all available scanning protocols. If the CBCT system had not been used in the past three hours, the X‐ray tube was warmed up with no filters or phantom in the beam before acquiring any images. Couch rails were out for all image acquisitions. The parameters for these protocols are given in Table 2, and all images were acquired using the reconstruction matrix size used clinically (384 pixels by 384 pixels).

All images were exported in DICOM format for offline analysis. Initial image analysis was attempted using an automated software (Automated CT Software (ACTS) v1.1; Gammex‐RMI), as well as manually using OsiriX (v.4.1.2, Pixmeo, Geneva, Switzerland). However, it was found that for the CT simulator images, the ACTS software was unable to perform automated analysis of some image sets due to an inability to detect the BBs used for localization. Since the ACTS software was not designed to analyze CBCT images, it was not surprising that some values obtained from the ACTS software did not agree with values obtained from manual analysis. With manual analysis, one could avoid placing regions of interest (ROIs) in locations with imaging artifacts, as seen in Fig. 3. Thus, it was decided to perform manual analysis alone for all subsequent image sets.

Except where noted, manual analysis of images was performed using ROI size and location, as well as analysis methods provided by the ACR.^1^, ^14^ ACR guidelines for image analysis also provide a reproducible method of evaluating images by suggesting standard window/level limits, so that analysis is not as subjective from month to month or user to user.^1^, ^14^ The image slices analyzed for the CT number (HU) accuracy and slice thickness module and the high‐contrast module were the slices in which the four alignment BBs were visible. For the uniformity and in‐plane distance accuracy module, the image slice analyzed included the two BBs for in‐plane distance measurement. For in‐plane distance measurements, a different window and level may be needed to visualize the BBs. For CBCT image analysis, the ROIs for HU and uniformity were placed so that they did not overlap with any imaging artifacts (e.g., the crescent artifact, as seen in Fig. 3). ROI sizes used for HU accuracy, low‐contrast resolution and uniformity, per ACR guidelines, are approximately $200\;{\text{mm}}^{2}$, $100\;{\text{mm}}^{2}$, and $400\;{\text{mm}}^{2}$, respectively.^(14)^ A template for ROI size and position was used in OsiriX for each imaging mode so that all ROI sizes and positions were consistent for all image sets.

For the low‐contrast module, the image slice approximately at the middle of the module with the best visualization of the low‐contrast cylinders on the CT simulator images was analyzed. Due to image noise in the CBCT images, the low‐contrast cylinders could not be distinguished from the background. Thus, in order to monitor the contrast‐to‐noise ratio (CNR) over time in the CBCT images, CNR was calculated for the polyethylene and acrylic plugs in the CT number accuracy and slice thickness module (Module 1) using a formula defined by the ACR:

(1) $$\text{CNR}=\left|{\text{ROI}}_{\text{mean}}-{\text{BACKGROUND}}_{\text{mean}}\right|/{\text{BACKGROUND}}_{\text{STD}}$$

where $ROI_{mean}$ is the mean HU value in the ROI for the plug, $BACKGROUND_{mean}$ is the mean HU in the ROI for the background, and $BACKGROUND_{STD}$ is the standard deviation in HU of the values in the ROI for the background. These two density plugs were used to provide a “low contrast” test since their accepted HU values are within ∼90−120 HU of the accepted HU value for the water plug. The water plug in the same slice was used for the background to provide a reproducibly placed background ROI from quarter to quarter. The CNR value for these two plugs was also calculated in this manner for the CT simulator images for comparison.

**Table 1:** Scanning parameters for the CT simulator protocols

	*Voltage (kVp)*	*Exposure (mAs)*	*Slice Thickness (mm)*	*Increment (mm)*	*Collimation (mm)*	*Display FOV (mm)*	*Scan FOV (mm)*	*Filter Type*	*Pitch*
Brain	120	500	3	3	16×0.75	600	300	UB	0.567
SRS Brain	120	500	1.5	1.5	16×0.75	600	289	UB	0.567
Pelvis	120	500	3	3	16×1.5	600	313	B	0.813
Chest	120	400	3	3	16×1.5	600	301	B	0.688
ENT	120	450	3	3	16×1.5	600	313	B	0.688

**Table 2:** Scanning parameters for the CBCT protocols on Varian linear accelerators

	*Voltage (kVp)*	*Exposure (mAs)*	*Gantry Rotation Range (°)*	*Number of Projections*	*Bowtie Filter*	*Slice Thickness (mm)*	*Fan Type*
Standard dose head	100	145	200	360	Full	2.5	Full
High‐quality head	100	720	200	360	Full	2.5	Full
Low‐dose head	100	72	200	360	Full	2.5	Full
Low‐dose thorax	110	262	360	655	Half	2.5	Half
Pelvis	125	680	360	655	Half	2.5	Half

Noise was also monitored for the polyethylene and acrylic plugs in the CT number accuracy and slice thickness module using the same ROIs placed for CNR and HU. Noise was calculated using the following formula:^9^, ^13^

(2) $$\text{Noise}(\%)=100\times {\text{ROI}}_{\text{STD}}/{\text{ROI}}_{\text{mean}}$$

where (in our case) $ROI_{mean}$ is the mean HU value in the ROI for the plug plus 1000 HU, and $ROI_{STD}$ is the standard deviation in HU of the values in the ROI the plug. The rescaling of the ${\text{ROI}}_{\text{mean}}$ values in the denominator of Eq. (2) was performed to prevent inflated noise values for plugs where the mean HU number was close to 0 HU and make our calculations comparable other publications.^9^, ^13^

## III. RESULTS

### A. CT Simulator

Image quality was within the ACR guidelines^1^, ^14^ (please see Recommendations and Conclusions section for a summary of these) for all of the scanning protocols tested. High‐contrast resolution was between 6–7 lp/cm for all of the CT simulator protocols tested. All measurements of slice thickness were within 0.5 mm of the nominal slice thickness. Most in‐plane distance measurements were within 0.5 mm, and all measurements were within 0.9 mm of the nominal 100.0 mm value.

**Figure 6:**
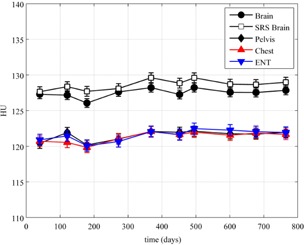
CT simulator HU over time for the acrylic plug in the ACR CT phantom. Error bars represent 1 SD over all measurements for a given scanning protocol.

All HU values were within the HU ranges provided by the ACR, except for the bone plug in the SRS and brain protocol images, which were within 10 HU of accepted values. The maximum standard deviation over time for all of the plugs was 1.1 HU. Figure 6 shows the HU values of the acrylic plug over time. The poorest uniformity value over all of the imaging protocols and measurements made was 2.9 HU, and the maximum standard deviation between uniformity values measured over time for all protocols was 0.7 HU. The HU value for the center circle in the uniformity module ranged from −3.5 to 5.1 HU, which was slightly above the ACR tolerance of ±5.0 HU.

CNR values in the low‐contrast module ranged from 1.02 to 2.05, with a maximum standard deviation of 0.21. All four of the 6 mm rods were visible on all of the images analyzed. Table 3 gives the average CNR and Table 4 gives the average noise over the two‐year measurement period for the polyethylene and acrylic plugs for each imaging mode.

**Table 3:** Average CNR and standard deviation over the two‐year measurement period for the polyethylene and acrylic plugs for each of the imaging modes on the CT simulator

	*Brain*		*SRS Brain*		*Pelvis*		*Chest*		*ENT*	
	*Avg*.	*SD*	*Avg*.	*SD*	*Avg*.	*SD*	*Avg*.	*SD*	*Avg*.	*SD*
Polyethylene	24.8	1.2	14.5	0.4	21.2	0.7	19.1	0.9	20.0	0.8
Acrylic	33.2	1.6	19.4	0.5	28.1	0.8	25.3	1.1	26.8	1.0

**Table 4:** Average noise and standard deviation over the two‐year measurement period for the polyethylene and acrylic plugs for each of the imaging modes on the CT simulator

	*Brain*		*SRS Brain*		*Pelvis*		*Chest*		*ENT*	
	*Avg. (%)*	*SD (%)*	*Avg. (%)*	*SD (%)*	*Avg. (%)*	*SD (%)*	*Avg. (%)*	*SD (%)*	*Avg. (%)*	*SD (%)*
Polyethylene	0.35	0.02	0.60	0.03	0.41	0.03	0.45	0.03	0.42	0.02
Acrylic	0.30	0.01	0.52	0.02	0.37	0.03	0.41	0.02	0.37	0.01

### B. CBCT

Since CBCT images are inherently noisier than CT images, ACR tolerance values are not applicable for all of the measurements, in particular HUs and uniformity. Initial measurements using the ACR CT phantom were used as baseline values for HUs for each density plug, and baseline values were also determined for high‐contrast resolution and uniformity. Preliminary results for HUs, high‐contrast resolution, and uniformity from the first three quarters of one CBCT system were used to establish preliminary tolerance values for comparison against baseline values. Subsequent results were monitored over the two‐year period using the preliminary tolerance values, and new baselines were acquired whenever major maintenance or recalibration was performed on the CBCT systems.

High‐contrast resolution for all three CBCT systems ranged from 4 lp/cm for half‐fan modes to 7 lp/cm for the full‐fan modes, and the maximum variation between baselines was 1 lp/cm over all three CBCT systems. All slice thickness measurements were within 0.5 mm of the nominal slice thickness. In‐plane distance measurements ranged from 99.1 mm to 100.8 mm, with a maximum standard deviation over all the measurements between baselines of 0.5 mm.

**Figure 7:**
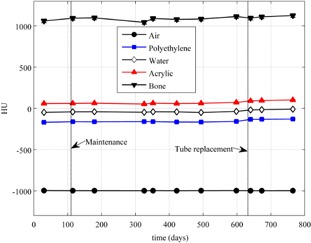
HU values for all of the density plugs in the ACR phantom for the 21EX‐B linac CBCT system over time for the standard dose head mode. Vertical lines indicate where maintenance and tube replacement occurred. Error bars fall within the data symbols and represent 1 SD over all measurements for each plug.

**Figure 8:**
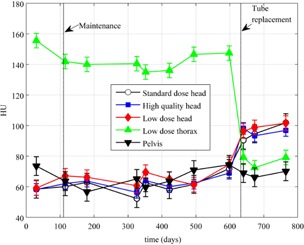
HU values for the acrylic plug over time for five of the imaging modes for the 21EX‐B CBCT system. Vertical lines indicate where maintenance and tube replacement occurred. Error bars represent 1 SD over all measurements for a given scanning protocol.

**Figure 9:**
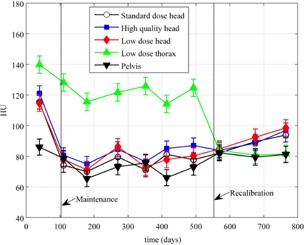
HU values for the acrylic plug over time for five of the imaging modes for the Novalis CBCT system. Vertical lines indicate where maintenance and recalibration occurred. Error bars represent 1 SD over all measurements for a given scanning protocol.

Figure 7 shows HU values as a function of time for all of the density plugs for one of the CBCT imaging modes. Figures 8 and 9 are plots of the HU values for the acrylic plug over time for five of the imaging modes for two of the CBCT systems. The maximum standard deviation for the different plugs between baselines for all of the CBCT systems and over all protocols used are as follows: air 3.1 HU, polyethylene 4.5 HU, water 7.9 HU, acrylic 6.1 HU, and bone 29.1 HU.

Uniformity values ranged from 15.1 to 24.0 HU for full fan modes and from 3.7 to 36.0 HU for half‐fan modes. The standard deviation of all the measurements for each mode between baselines was determined for all three CBCT systems, and the maximum standard deviation for all modes across all three CBCT systems was 11.0 HU.

Due to the extra noise in CBCT images from scattered photons, the low‐contrast plugs in the CBCT module could not be distinguished from the background. Thus, the CNR using the polyethylene and acrylic plugs with the water plug as the background was calculated. Table 5 gives the average CNR, and Table 6 gives the average noise and standard deviation for these two plugs between baselines for each CBCT system.

**Table 5:** Average CNR and standard deviation from baseline to baseline for each CBCT system

		*Std. Dose Head*		*Hi‐quality Head*		*Low‐dose Head*		*Low‐dose Thorax*		*Pelvis*	
		*Avg*.	*SD*	*Avg*.	*SD*	*Avg*.	*SD*	*Avg*.	*SD*	*Avg*.	*SD*
Novalis	Polyethylene	3.4	0.1	7.4	0.2	4.2	1.8	11.8	0.6	12.1	1.1
	Acrylic	3.5	0.0	7.5	0.3	4.2	1.6	12.9	0.9	14.2	1.2
21EX‐A	Polyethylene	4.0	0.3	7.3	0.4	4.3	0.2	10.4	0.7	10.0	1.3
	Acrylic	3.2	0.2	6.0	0.4	3.4	0.2	10.7	1.1	11.0	1.5
21EX‐B	Polyethylene	3.7	0.2	6.6	0.3	4.0	0.6	9.9	0.3	9.3	0.4
	Acrylic	3.3	0.1	6.1	0.3	3.7	0.6	9.3	0.4	9.3	0.2

**Table 6:** Average noise and standard deviation from baseline to baseline for each CBCT system

		*Std. Dose Head*		*Hi‐quality Head*		*Low‐dose Head*		*Low‐dose Thorax*		*Pelvis*	
		*Avg. (%)*	*SD (%)*	*Avg. (%)*	*SD (%)*	*Avg. (%)*	*SD (%)*	*Avg. (%)*	*SD (%)*	*Avg. (%)*	*SD (%)*
Novalis	Polyethylene	3.5	0.11	1.9	0.08	3.7	0.06	1.04	0.06	0.87	0.07
	Acrylic	3.2	0.06	1.5	0.05	3.1	0.09	1.05	0.08	0.9	0.06
21EX‐A	Polyethylene	3.9	0.20	2.7	0.29	4.2	0.37	1.0	0.07	1.10	0.07
	Acrylic	3.3	0.14	1.9	0.09	3.3	0.43	1.09	0.07	1.0	0.08
21EX‐B	Polyethylene	3.7	0.09	2.0	0.10	3.3	0.42	1.1	0.04	1.14	0.05
	Acrylic	3.4	0.18	1.7	0.10	3.1	0.40	1.21	0.06	1.1	0.05

## IV. DISCUSSION

### A. CT simulator

Even after maintenance, the CT simulator images should still be within ACR guidelines. However, the ACR tolerance values were not published in a peer reviewed journal since they are “energy dependent and the manufacturers filter their beams differently”, and the values “were set early in the ACR CT Accreditation Program … based on experiences of the physics subcommittee” (R. Magosing, email correspondence regarding question about tolerance values for ACR CT phantom, March 27, 2013). Since some of the ACR tolerances are quite loose (see Table 7), a trend in measurements that could indicate decreasing image quality could be missed using them. Therefore, statistical analysis of the CT simulator values was performed using all available measurements in order to investigate whether any of the ACR tolerances should be adjusted. Our method was based on that suggested in Pawlicki et al.^(15)^ for statistical process control, with the goal with our analysis and adjusted tolerance levels to indicate a degradation in machine performance, as opposed to an inability to use the system clinically. For several of the measured parameters, our method assumed that the fluctuations in results from quarter to quarter could be characterized using the standard deviation across the results, and tolerance levels were then set at two times the standard deviation. Any individual result outside the tolerance level would be expected to occur only about 5% of the time, assuming that the data are normally distributed (which was not tested for in this analysis). If an individual result was outside the tolerance level, this would warrant further investigation to determine if the machine performance had degraded. Other tolerance levels were set based on the worst‐case results over the two‐year measurement period.

**Table 7:** ACR tolerance values and additional recommendations from authors

		*Additional Recommendations From Authors*	
	*ACR*	*CT Simulator*	*CBCT*
High‐contrast resolution	5 lp/cm for adult abdomen, 6 lp/com for high‐resolution chest	6 lp/cm	±1 lp/cm from baseline measurement
Slice thickness	±1.5 mm of nominal slice thickness	±0.5 mm of nominal	±0.5 mm of nominal
In‐plane distance	‐	±1.0 mm of nominal 100.0 mm value	±1.0 mm of nominal 100.0 mm value
			Air: ±6 HU
			Polyethylene: ±9 HU
HU values	Recommended ranges differing from each plug	±3 HU from baseline measurement	Water: ±16 HU
			Acrylic: ±12 HU
			Bone: ±12 HU
Uniformity	Must be <5 HU for all four edge positions, center region must be between ±7 HU	±1.4 HU of baseline measurement	±22 HU of baseline measurement
CNR: Contrast module	All four 6 mm rods visible, CNR minimum of 1.0	Minimum of 1.0, within 0.4 of baseline values	‐
CNR: polyethene and acrylic plugs	‐	±3.2 and ±2.4 of baseline measurements for acrylic and polyethylene, respectively	±3.3 and ±3.6 of baseline measurements for acrylic and polyethylene, respectively
Noise	‐	0.07% and 0.05% of baseline measurements for polyethylene and acrylic plugs, respectively	0.84% and 0.87% of baseline measurements for polyethylene and acrylic plugs, respectively

ACR recommendations for high‐contrast resolution are a minimum of 5 lp/cm for an adult abdomen scan and 6 lp/cm for a high‐resolution chest scan.^(1)^ Since our results showed that the minimum high‐contrast resolution observed was 6 lp/cm, our minimum tolerance should be changed to 6 lp/cm. Given that all slice thicknesses measured were within 0.5 mm of the nominal slice thickness, our tolerance should be decreased to 0.5 mm. For the in‐plane distance measurement, there are no specific ACR tolerances, but from our measurements we propose a 1.0 mm tolerance.

Typical tolerances for radiotherapy centers water HU range and field uniformity on CT simulators are from −5 to +5 HU and 5 HU, respectively,^(2)^ which are also what is suggested by the ACR.^1^, ^14^ In view of the fact that the accepted ACR HU ranges are quite large for some of the plugs in comparison with the consistency in our results, we propose tighter tolerances on HU values and maximum uniformity after baseline measurements are established. HU values should be within ±3 HU (approximately twice the maximum standard deviation) of baseline HU values for each of the plugs. If the HU values drift away from ±3 HU of baseline values, this could possibly indicate a change in the energy of the beam, which can affect image quality. Since our maximum uniformity measurement was 2.9 HU, in addition to ensuring that the ACR guideline of 5 HU is met, uniformity measurements should also fall within ±1.4 HU of baseline uniformity measurements.

Because the CNR values in the low‐contrast module ranged from 1.0 to 2.0, the minimum ACR value of 1.0 is acceptable. Based on our two‐year data, an additional tolerance ensuring that these values stay both above 1.0 and within 0.4 of baseline values will make sure that the low‐contrast resolution does not degrade over time. ACR tolerance values do not exist for our additional CNR test, including the acrylic and polyethylene plugs. These CNR values should stay within ±3.2 and ±2.4, respectively, of baseline measurements for acrylic and polyethylene.

From Table 4, the largest average noise is measured when using the SRS brain mode. This is not surprising, since this mode has the smallest slice thickness. From our analysis, the tolerance on the noise in the polyethylene and acrylic plugs should be 0.07% and 0.05%, respectively, when compared to baseline noise measurements.

### B. CBCT

All of the preliminary tolerance values were refined with analysis of data between baseline measurements over the two‐year measurement period, and typical tolerance values for a parameter were set at twice the maximum standard deviation over all of the imaging protocols and all of the CBCT imaging systems. These generic tolerances are only applicable since we have three CBCT systems that are nominally the same in our department. More thorough error detection, such as that suggested by Pawlicki et al.,^(15)^ would provide quantitative tolerance values that are specific to both a protocol and an individual CBCT system. We have chosen to use a method based on standard deviations because it provides a simpler way to routinely analyze our data and provide a quantitative indication of changes in image quality parameters of our CBCT systems.

Since the maximum deviation from baseline to baseline for the high‐contrast resolution was 1 lp/cm, the tolerance used should be ±1 lp/cm from baseline measurements. Other authors have reported a minimum high‐contrast resolution of 5‐7 lp/cm for CBCT systems,^6^, ^12^ but these results seem to not be for all available imaging modes. Given that each mode has different acquisition settings, each mode should be tested since a decrease in high‐contrast spatial resolution from baseline could point out a change in the CBCT geometry or calibration.^(6)^

Slice thickness results were similar to results from the CT simulator; therefore, the tolerance should be the same at ±0.5 mm of nominal slice thickness. Using the maximum standard deviation of in‐plane distance measurements between baselines for all three CBCT systems, in‐plane distance measurements should fall within ±1.0 mm of the nominal 100.0 mm value.

It can be observed that the HU values seen in Fig. 6 for the acrylic plug on the CT simulator are not close to the values seen in Figs. 8 and 9 on two of the CBCT systems. One explanation for this is that the CBCT system is calibrated using the Catphan, which is composed of different materials than the ACR phantom. Since CBCT HUs are sensitive to differences in scatter sources, scanning mode (half‐fan versus full‐fan), and phantom size^5^, ^8^, ^11^, ^16^, ^17^, ^18^ and reconstruction methods potentially do not provide adequate beam hardening and scatter correction methods,^(19)^ these differences in HU values between CBCT and CT simulator measurements are not surprising and lead to the necessity for separate tolerances to be developed.

Figures 8 and 9 also demonstrate that, before the tube replacement and recalibrations, the HU values on these two CBCT systems had drifted away from each other or initially were not properly calibrated. Once the recalibrations were performed, HU values for the acrylic plug for the half‐fan measurements were within ∼20−30 HU of full‐fan measurements. It can also be observed from Figs. 8 and 9 that the HU values measured on the two different CBCT systems for the acrylic plug were quite similar. Similar results were seen for the other plugs when comparing HU values between CBCT systems.

Thus, HU tolerance values were set to twice the maximum standard deviation over all three CBCT systems over all protocols for each plug and rounded to the nearest HU. These tolerance values are listed in Table 7 and are used to compare quarterly HU measurements to baseline HU measurements. These tolerance values are much smaller and more specific than the general tolerance value of ±40 HU suggested by other authors and vendors.^7^, ^10^, ^11^, ^12^

Since this QA analysis uses only one image and one phantom orientation for HU reproducibility, these numbers should not be used for generating HU to electron density curves for dose calculations with CBCT images. If dose calculations using CBCTs need to be performed, HU values should first be determined using a phantom with more inserts than the ACR phantom, with a diameter close to that of the phantom/patient that dose calculations will be performed, with multiple phantom orientations, and from multiple image slices of that phantom.^5^, ^8^, ^11^, ^16^, ^17^, ^18^, ^20^, ^21^, ^22^, ^23^

The wide range of uniformity values in the CBCT measurements as compared to the CT simulator measurements is indicative of the known nonuniformity in CBCT images. This nonuniformity is due to difficulties in image reconstruction due to scattering and beam hardening which cause the cupping artifact, the reduction in brightness at the center of the image.^17^, ^18^ For uniformity measurements, a baseline value was set, and subsequent measurements should fall within ±22 HU of baseline results.

CNR values for the polyethylene and acrylic plugs are quite similar between the three CBCT systems, indicating that “low” contrast objects should be seen just as well if a patient has to be moved from one linac to the other. The maximum standard deviations between baselines over all CBCT systems and imaging protocols for polyethylene and acrylic give tolerance values of ±3.6 and 3.3, respectively, from baseline CNR measurements. CNR also increases with mAs, as indicated by the increased CNR values of the high‐quality head, pelvis, and low‐dose thorax modes, compared to the other three modes. These results are not truly indicative of “low” contrast resolution for the pelvis and low‐dose thorax modes since the diameter of the ACR CT phantom is smaller than any patient's pelvis or thorax.

In comparing the noise values in Tables 4 and 6, it can be observed again that the noise in the CBCT images is greater than in the CT simulator images. From our analysis, the tolerance on the noise in the polyethylene and acrylic plugs should be 0.84% and 0.87%, respectively, when compared to baseline noise measurements.

### C. Phantom improvements

A number of improvements to the ACR phantom could be made to make it more amenable to real patient scanning conditions, as well as provide the optimal testing for both CT simulator and CBCT imaging. One drawback of the current ACR phantom design is the inability to visualize the low‐contrast inserts in the low‐contrast module on Varian CBCT systems. Yoo et al.^(12)^ reported that for 1% contrast difference, 7 mm disks could be distinguished from background. Since the ACR phantom currently has 0.6% contrast difference from the background in the low‐contrast resolution module,^(1)^ our suggestion is to split the low‐contrast resolution module into two — one with 0.6% for CT simulators and one with 1% for CBCT.

Also, as previously mentioned, the ACR CT phantom is closer in diameter to an average head than a body, so it is not close to imitating scattering and beam hardening conditions for extracranial sites. Future work would use the outer shell that is supposed to mimic pelvis or thorax conditions, to monitor the image quality parameters under these conditions.

## V. RECOMMENDATIONS & CONCLUSIONS

The value of using the ACR phantom is that it allows one to use quantitative methods for monitoring image quality and provides established tolerance values that can be used as an initial goal for CBCT image quality QA. An additional value is that we are currently using one phantom to measure image quality parameters on both our CBCT systems and CT simulator. However, since our analysis requires initial manual placement of ROIs and distance measurements, it could be considered quite time‐consuming to implement this procedure, especially if a center has multiple CBCT imaging systems. At the time of publication, automated software such as Gammex's ACTS for the ACR CT phantom and Image Owl software for the Catphan, however, is not able to automatically analyze CBCT images due to the additional noise present in these images. Once these programs have been improved to be able to analyze CBCT images, users could use these for the majority of the measurements. However, in establishing tolerance values for each of the tests, we suggest that long‐term analysis, such as presented in this article, still be performed in order to determine whether the tolerance values used in these programs can be universally applied to all CBCT systems, as well as to provide a better monitoring of the image quality parameters.

Table 7 can be used as a guideline for initial tolerance values for a center starting to use the ACR CT phantom on CT simulators or linac CBCTs manufactured by the same vendors as our center. For other centers with CT simulators or linac CBCTs manufactured by different vendors, we have proposed a methodology that could be used for setting tolerance values. After long‐term data have been acquired, similar analysis should be performed to see if these suggested tolerance values are adequate or if the tolerances need to be increased or decreased depending on the individual CBCT system. Future measurements outside these tolerance levels can then be used as a possible early indication of machine degradation, before the system reaches a point where it could have a negative impact on patient care.
