# Erratum: Using the ACR CT accreditation phantom for routine image quality assurance on both CT and CBCT imaging systems in a radiotherapy environment

**DOI:** 10.1120/jacmp.v15i6.5228

**Published:** 2014-11-08

## ORIGINAL CITATION

Hobson MA, Soisson ET, Davis SD, Parker W. Using the ACR CT accreditation phantom for routine image quality assurance on both CT and CBCT imaging systems in a radiotherapy environment. J Appl Clin Med Phys. 2014;15:226–39.

In this erratum, we present the corrections for a typographical error that occurred in the above‐noted publication in Table 7 on page 235. The CBCT HU tolerance value for bone should be ±58HU, and the corrected table is below.

**Table 7:** ACR tolerance values and additional recommendations from authors.

		*Additional Recommendations From Authors*	
	*ACR*	*CT Simulator*	*CBCT*
High‐contrast resolution	5 lp/cm for adult abdomen, 6 lp/cm for high‐resolution chest	6 lp/cm	±1lp/cm from baseline measurement
Slice thickness	±1.5mm of nominal slice thickness	±0.5mm of nominal	±0.5mm of nominal
In‐plane distance	–	±1.0mm of nominal 100.0 mm value	±1.0mm of nominal 100.0 mm value
			Air: ±6HU
			Polyethylene: ±9HU
HU values	Recommended ranges differing from each plug	±3HU from baseline measurement	Water: ±16HU
			Acrylic: ±12HU
			Bone: ±58HU
Uniformity	Must be <5HU for all four edge positions, center region must be between ±7HU	±1.4HU of baseline measurement	±22HU of baseline measurement
CNR: Contrast module	All four 6 mm rods visible, CNR minimum of 1.0	Minimum of 1.0, within 0.4 of baseline values	–
CNR: polyethene and acrylic plugs	–	±3.2 and ±2.4 of baseline measurements for acrylic and polyethylene, respectively	±3.3 and ±3.6 of baseline measurements for acrylic and polyethylene, respectively
Noise	–	0.07% and 0.05% of baseline measurements for polyethylene and acrylic plugs, respectively	0.84% and 0.87% of baseline measurements for polyethylene and acrylic plugs, respectively

